# Prefrontal fNIRS hemodynamic correlates of attentional load during rapid serial visual presentation tasks

**DOI:** 10.3389/fnhum.2026.1843879

**Published:** 2026-06-08

**Authors:** Seongyeon Lim, Suh-Yeon Dong, Tzyy-Ping Jung

**Affiliations:** 1 Department of Information Technology Engineering, Sookmyung Women’s University, Seoul, Republic of Korea; 2 Division of Artificial Intelligence Engineering, Sookmyung Women’s University, Seoul, Republic of Korea; 3 Swartz Center for Computational Neuroscience, Institute for Neural Computation, University of California, San Diego, CA, United States

**Keywords:** attentional monitoring, functional connectivity, functional near-infrared spectroscopy (fNIRS), hemodynamic response, prefrontal cortex, rapid serial visual presentation (RSVP)

## Abstract

**Background:**

Despite the growing interest in functional near-infrared spectroscopy (fNIRS) for practical brain-computer interface (BCI) applications, prefrontal hemodynamic responses during rapid serial visual presentation (RSVP) tasks remain poorly characterized, even though these tasks demand sustained attentional engagement under fast-paced visual streams.

**Methods:**

We examined prefrontal cortex (PFC) activation and functional connectivity as indices of attentional monitoring during an RSVP task using a 15-channel prefrontal fNIRS device in 50 participants. Trials either contained one target image among nontargets or consisted entirely of nontarget images. Eleven statistical activation features from oxygenated (HbO) and deoxygenated (HbR) hemoglobin changes, and Fisher’s r-to-z transformed inter-channel connectivity values were compared between conditions using paired-samples t-tests with false discovery rate correction. Response time and exploratory correlations between behavioral latency and hemodynamic features were also analyzed.

**Results:**

The nontarget condition showed slightly higher HbO activation, mainly in amplitude-related features such as max and mean, suggesting increased sustained processing demands when target absence had to be confirmed. In contrast, HbR differences were more strongly characterized by distributional and transient-related features, including kurtosis, skewness, and peak-related features, suggesting complementary HbO and HbR sensitivity to task conditions. Connectivity analysis revealed condition-dependent inter-channel coupling patterns, with generally stronger HbO connectivity in the nontarget condition and a partially different HbR pattern. Response times were significantly longer in the nontarget condition and were more closely associated with temporal and distributional hemodynamic features than with amplitude-based features.

**Conclusion:**

These findings provide an initial exploratory characterization of condition-dependent prefrontal activation and connectivity differences during RSVP tasks, highlighting the potential of fNIRS as a practical tool for attentional monitoring and informing future multimodal neuroimaging approaches.

## 1 Introduction

The rapid serial presentation (RSVP) paradigm presents visual stimuli in quick succession, requiring sustained attentional engagement to monitor a rapid stream of images for a designated target (Chun and Potter, 1995). Owing to its ability to probe rapid shifts of attention and target monitoring processes (Broadbent and Broadbent, 1987; Lees et al., 2018; Xu et al., 2025), RSVP has been widely applied in visual surveillance, security, and military operations, as well as in human-computer interaction (HCI) and brain-computer interface (BCI) research (Chun and Potter, 1995; Broadbent and Broadbent, 1987).

Electroencephalography (EEG) has traditionally dominated RSVP-BCI studies because of its millisecond-level temporal resolution for capturing rapid attentional fluctuations (Zhang et al., 2020; Lian et al., 2023). However, practical BCIs also require portable and motion-resistant neuroimaging tools suitable for use outside controlled laboratory settings (Klein et al., 2024; Boas et al., 2024; Finnis et al., 2025). In this regard, functional near-infrared spectroscopy (fNIRS), owing to its non-invasiveness, portability, and relative robustness to motion artifacts, has increasingly attracted attention as a practical neuroimaging modality for BCI applications (Finnis et al., 2025; Paulmurugan et al., 2021). Although the inherently slow hemodynamic response of fNIRS limits its sensitivity to rapid event-level neural dynamics, it is well suited for capturing sustained, sequence-level attentional responses that unfold over several seconds (Paulmurugan et al., 2021). Characterizing prefrontal hemodynamic patterns during cognitively demanding tasks is therefore essential for establishing the utility of fNIRS in practical attentional monitoring applications beyond conventional laboratory settings (Klein et al., 2024; Krelove and Mochizuki, 2025; Zapała et al., 2022). Yet to date, no study has systematically characterized prefrontal hemodynamic responses during RSVP tasks using fNIRS, leaving this gap unaddressed.

Among the cortical regions accessible to fNIRS, the prefrontal cortex (PFC) is particularly relevant for RSVP-based attentional monitoring, as it supports sustained attention, working memory, and cognitive control, which are also engaged during RSVP-based tasks (Fan et al., 2021; Yan et al., 2025; Gemmerich et al., 2025). These functions are closely related to RSVP task demands, including the monitoring of task-relevant targets during rapid visual streams (Bellet et al., 2022; Zhou and Geng, 2025) and the inhibition of responses to frequent nontarget stimuli (Friedman and Robbins, 2022; Benn and Robinson, 2024). In addition, target and nontarget conditions may impose different attentional demands. Although fNIRS has been used to investigate PFC activation alongside inter-channel functional connectivity in various cognitive tasks (Yan et al., 2025; Gemmerich et al., 2025), studies examining both activation and connectivity during fast-paced visual stream paradigms such as RSVP remain underexplored (Xu et al., 2025; Pinto-Orellana et al., 2024). Understanding how the PFC modulates its hemodynamic responses across different attentional demands in these contexts is therefore necessary to advance fNIRS-based attentional monitoring and inform future multimodal BCI system design.

Previous fNIRS studies using cognitively demanding tasks such as working memory and executive control have consistently demonstrated activation and network changes in the PFC, suggesting that incorporating sufficient post-stimulus rest periods may also enable stable hemodynamic capture in temporally demanding visual paradigms such as RSVP (Guevara et al., 2024; Baker et al., 2018; Chen et al., 2022). As an initial exploration of prefrontal fNIRS responses under rapid visual attention demands, we hypothesized that target and nontarget conditions would elicit distinct prefrontal activation and connectivity patterns, reflecting differences in attentional demands imposed by each condition. Characterizing these differences was expected to provide a preliminary physiological basis for fNIRS-based attentional monitoring in future multimodal BCI applications.

## 2 Materials and methods

### 2.1 Participants

Fifty healthy adults (all females; mean age ± standard deviation: 23.6 ± 2.65 years) participated in this study. Individuals with a history of neurotic disorders or heart disease, those currently using antidepressants, anticonvulsants, or sleeping aids in the past month, and those using medications affecting the endocrine system, along with pregnant individuals or those planning pregnancy, were not included in this study. All participants received verbal instructions regarding the entire experimental procedure, and written informed consent was obtained. The Institutional Review Board of Sookmyung Women’s University approved this study (IRB No. SMWU-2407-HR-054).

### 2.2 Experimental paradigm

We used vehicle images from the STL-10 dataset (Coates et al., 2011). Images were selected from the airplane, car, ship, and truck classes, excluding those with irrelevant features, partially obscured objects, or excessively large objects. A single airplane image, showing a red airplane against a sky background, was designated as the target and was consistently used across all target trials. Thus, the target image did not change between trials. Nontarget images were randomly selected from the remaining transportation images, resulting in a pool of 720 images. The image size was increased to 288 × 288 pixels to enhance visual clarity. To minimize background interference with object recognition, objects were isolated from their background using the Python library Rembg (Gatis, 2025), and a Gaussian filter was applied to blur the backgrounds. For the stimuli, we created 40 image sequences, of which 20 were target sequences. Each sequence comprised 20 randomly selected images. Target sequences contained exactly one target image and 19 nontarget images, while the nontarget sequence consisted entirely of nontarget images. The position of the target image within each sequence was randomized to prevent temporal expectation effects and to introduce variability in stimulus timing across trials. The stimuli were presented to participants using PsychoPy (Peirce, 2007).

**Figure 1:**
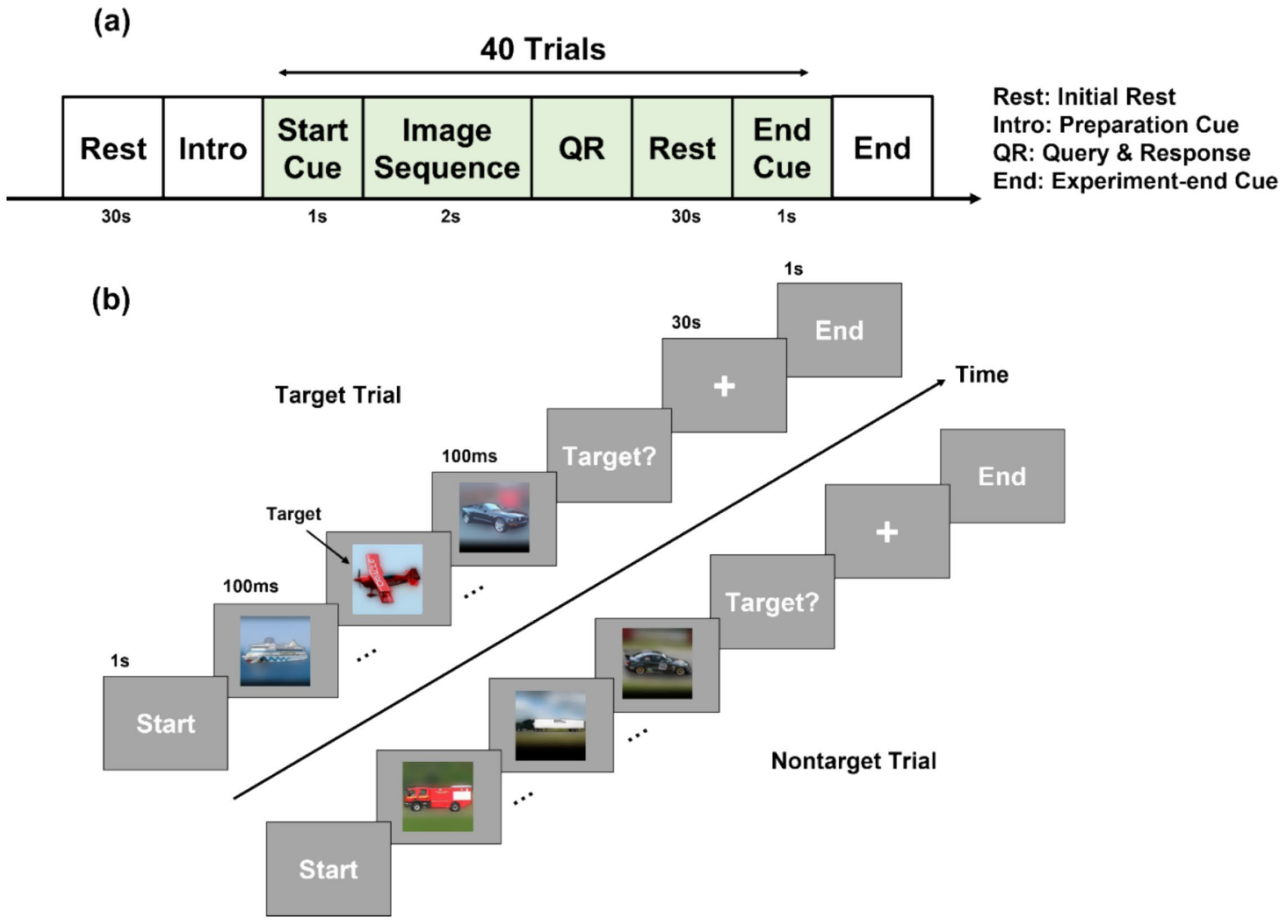
Experimental paradigm. **(a)** Overall experimental timeline. After a 30 s initial rest, the “Intro” indicates a user-initiated preparation cue with no fixed duration, followed by 40 RSVP trials, and “End” denotes the termination of the experiment. **(b)** Structure of a single target or nontarget trial. Each trial began with a 1 s “Start” cue, a 2 s image sequence presented at 100 milliseconds per image. The position of the target stimulus within each target sequence was randomized to reduce temporal expectation effects during target-present trials. This was followed by a self-paced query and response phase, after which the trial immediately proceeded to a 30 s resting period, and ended with a 1 s “End” cue after fixation. The example stimulus images shown in the figure were selected from the STL-10 dataset (Coates et al., 2011).

Figure 1a illustrates the overall experimental timeline. Participants first completed a 30-s initial resting period to establish a baseline. fNIRS signals were recorded only during the subsequent RSVP trials, excluding the initial baseline period. Before the first trial, a readiness prompt was displayed, and participants initiated the task by pressing the spacebar. Participants then completed 40 RSVP trials, randomly assigned as either target or nontarget trials, as shown in Figure 1b. Each trial comprised a sequence of events beginning with a 1-s “Start” cue, followed by 20 rapidly presented images over 2 s at a rate of 100 milliseconds per image. After the stimulus presentation, participants entered a query and response phase, during which participants indicated whether a target was present by pressing the “0” or “1” key. The response phase was self-paced, and the trial proceeded immediately to the subsequent resting period upon the participant’s response, resulting in variable inter-trial intervals. No formal temporal jitter was introduced between trials. However, temporal variability was introduced through randomized target positions within target sequences, randomized presentation of target and nontarget trials, and the self-paced response phase. Following the response, a 30-s resting period was included to account for the delayed hemodynamic response, and each trial ended with a 1-s “End” cue after fixation on a central cross. The experiment concluded after participants completed all 40 trials.

### 2.3 Data acquisition

**Figure 2:**
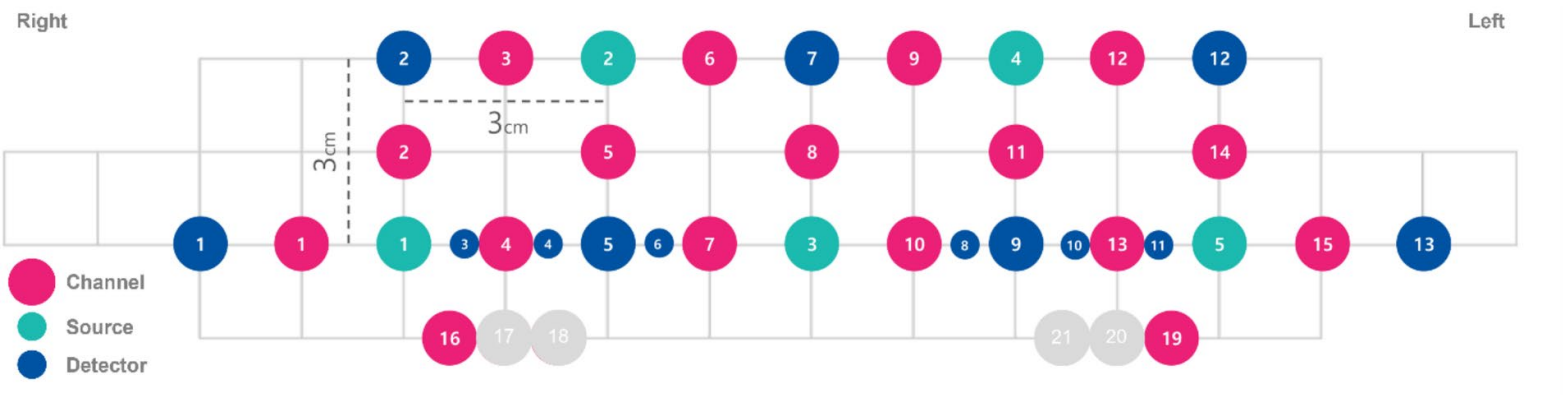
Prefrontal channel layout of the NIRSIT Lite device. Ch01–Ch15 indicated 3 cm long-separation measurement channels used for activation and functional connectivity analyses. Ch16 and Ch19 indicate 8 mm short-separation channels used only for superficial physiological signal regression. Gray channels indicate channels not used in the present analysis. The layout was adapted from the NIRSIT Lite manual from OBELAB Inc.

We used a 15-channel NIRSIT Lite device (OBELAB Inc., Seoul, Korea) to record hemodynamic responses from the PFC. The device is a wearable headband-type system with a fixed optode arrangement designed to cover the PFC. It was positioned on the participant’s forehead and aligned according to the international 10–10 system, with the central optodes approximately corresponding to the AFz region. Facial landmarks, including the eyes, nose, and eyebrows, were used to maintain midline placement across participants. The forehead region was cleared of hair when necessary to ensure proper optode-scalp contact, and the headband was secured and adjusted using a strap to maintain a stable and comfortable fit. Prior to data acquisition, a calibration procedure was performed to verify signal quality and ensure proper optode-scalp contact. Optode locations were not digitized. Instead, standardized placement based on the 10–10 system was used across participants. The device comprises 5 sources, 13 detectors, forming 15 long-separation measurement channels, Ch01-Ch15, with a source-detector distance of 3 cm. Ch16 and Ch19 are 8 mm short separation channels and were used only for superficial physiological signal regression. Ch17, Ch18, Ch20, and Ch21 were not used in the present analysis. Near-infrared light at 750 nm and 850 nm was used to estimate concentration changes in HbO and HbR, respectively. Data were acquired using NIRSIT SCAN v1.3.1 software at a sampling rate of 8.138 Hz. Figure 2 shows the prefrontal channel layout of the NIRSIT Lite device (Kim et al., 2022).

### 2.4 Data preprocessing

We preprocessed the fNIRS data using the NIRSIT QUEST v1.1.3 software. The preprocessing pipeline consisted of channel quality control, signal conversion, motion correction, physiological noise removal, baseline correction, and filtering. Raw light intensity signals were screened to remove unreliable channels. Channels were excluded if they contained invalid intensity values, showed a median intensity below 30, had a coefficient of variation greater than 15 percent, or exhibited signal saturation defined as constant values persisting for more than 5 percent of the time series (Yücel et al., 2021; Pfeifer et al., 2018). The remaining intensity signals were converted into optical density. Motion artifacts were corrected at the optical density level using the Temporal Derivative Distribution Repair (TDDR) method (Fishburn et al., 2019). To reduce contamination from superficial physiological signals such as scalp blood flow, short channel regression was applied (Yücel et al., 2021). Specifically, the first principal component was derived from the two 8 mm short separation channels, Ch16 and Ch19, and used as a representative superficial physiological regressor. This regressor was removed from each of the 15 long separation channel signals before subsequent HbO and HbR conversion and analysis (Gagnon et al., 2011). Optical density signals were converted into concentration changes of HbO and HbR using the modified Beer–Lambert law (Delpy et al., 1988; Cope and Delpy, 1988). Molar extinction coefficients followed (Zhao et al., 2017). A baseline correction was applied by defining the interval from 2 s before stimulus onset to stimulus onset as the reference period and normalizing the signals relative to this baseline. No additional zero-mapping or *z*-scoring across participants was applied. Instead, HbO and HbR concentration changes were analysed relative to the pre-stimulus baseline. The resulting haemoglobin signals were band-pass filtered between 0.005 Hz and 0.1 Hz to remove physiological noise and slow drift components (Yücel et al., 2021). Additional channel rejection excluded signals showing a strong negative correlation between HbO and HbR below −0.9, indicating potential artifacts (Takizawa et al., 2014). On average, 1.2 channels per participant were excluded during processing, corresponding to an overall rejection rate of 8 percent across all channels. For each trial, only the first 18 s of the post-stimulus rest period were used for analysis. This interval begins immediately after stimulus offset and captures the task-evoked hemodynamic response based on prior findings (Pinti et al., 2020; Yang et al., 2020; Pinti et al., 2021). The remaining portion of the rest period was excluded to allow sufficient physiological recovery between trials.

### 2.5 Data analysis

To capture both time-domain and network-level properties of prefrontal hemodynamic responses, we performed two complementary analyses, activation feature extraction and functional connectivity analysis. Only trials with correct RSVP responses were included in the analysis, as incorrect responses may indicate lapses in attention rather than sustained attentional processing, which is the focus of the present study. Of the 2,000 trials completed across all participants, four trials were excluded due to incorrect responses, resulting in 1,996 trials in the final analysis.

The selected features were designed to capture complementary aspects of the hemodynamic response relevant to attentional processing. Specifically, amplitude-based features such as mean, max, and min reflect overall activation levels (Afzal Khan and Hong, 2021). Variability-related features, including variance, standard deviation, and slope, characterize temporal fluctuations in the hemodynamic signal (Naseer and Hong, 2015; Naseer et al., 2016). Higher-order statistical and peak-based features, including skewness, kurtosis, peak_sum, peak_mean, and peak_max, capture transient and asymmetric response dynamics associated with event-related neural engagement (Khan and Hong, 2015). Peak-based features were calculated as follows. Local peaks within each epoch were identified as data points whose amplitude was greater than that of their immediately adjacent samples. The amplitudes of all detected peaks were then extracted, and peak_sum and peak_max were computed as the sum and maximum of those peak amplitudes, respectively (Khan and Hong, 2015). Additionally, peak_mean was defined as the arithmetic mean of the detected peak amplitudes, providing a complementary measure of average peak-level activation across the epoch. These feature categories have been widely employed in prior fNIRS studies to characterize task-evoked PFC hemodynamic responses (Afzal Khan and Hong, 2021; Naseer and Hong, 2015; Naseer et al., 2016; Khan and Hong, 2015). Given the relatively large number of features and channels, multiple comparisons were conducted across features and channels to systematically assess the discriminative contribution of each feature category and to identify which hemodynamic characteristics most reliably differentiate attentional states, rather than relying on a single composite measure (Naseer et al., 2016; Khan and Hong, 2015). This analysis was conducted in an exploratory manner to comprehensively assess the discriminative contribution of each feature category across channels.

For the functional connectivity analysis, Pearson’s correlation coefficients were calculated between all possible pairs of the 15 channels using the HbO and HbR time series, with connectivity computed separately for each signal (Nguyen et al., 2018; Nguyen et al., 2019; Ye et al., 2024; Novi et al., 2023). For each trial, correlations were computed using the full time series within a 21-s epoch (Nguyen et al., 2018), resulting in a 15 × 15 channel-wise correlation matrix (Eng et al., 2025). The upper triangular elements, excluding the diagonal, were extracted to avoid redundancy (Novi et al., 2023), and normalized using Fisher’s r-to-z transformation to improve normality (Nguyen et al., 2019; Eng et al., 2025).

In addition, response time (RT) was recorded for each trial as a behavioral measure, defined as the interval from the onset of the query prompt to the participant’s key press. To investigate the relationship between hemodynamic features and behavioral performance, Pearson’s correlation coefficients were computed between the mean RT and each activation feature at the participant level. RT and feature values were averaged across 20 trials within each condition per participant prior to correlation analysis (Memmini et al., 2020). Given the exploratory nature of this analysis, correlations with *p* < 0.05 were considered nominally significant without correction for multiple comparisons (Pinti et al., 2020).

### 2.6 Statistical analysis

Paired-sample t-tests were conducted to compare activation and connectivity features between target and nontarget conditions (Pinti et al., 2020; Field, 2024). Before statistical testing, each participant’s feature values were averaged across 20 trials within each condition, yielding one representative value per participant per condition (degrees of freedom, *df* = 49). This participant-level approach was adopted to reflect the within-subject design of the study, as each participant was exposed to both target and nontarget conditions. Response times between conditions were also compared using a paired-sample *t*-test at the participant level. All *t*-tests were corrected for multiple comparisons using the Benjamini-Hochberg FDR correction (Benjamini and Hochberg, 1995), and results were considered statistically significant at corrected *p*-values (*p*) below 0.05. Effect sizes were reported using Cohen’s d ( d ), where d = 0.2 indicates a small effect, d = 0.5 a medium effect, and d = 0.8 a large effect (Cohen, 2013). All values are reported as mean ± standard deviation (SD), unless otherwise specified. All statistical analyses were performed in Python 3.11.3 using the SciPy Stats 1.10.1 package.

## 3 Results

### 3.1 Behavioral results

Response times were significantly longer in the nontarget condition (0.803 ± 0.468 s) than in the target condition (0.703 ± 0.441 s), indicating longer response latency in the absence of a target (*t* = −4.801, *p* < 0.001, d = 0.679).

### 3.2 Relationship between response time and hemodynamic features

To investigate the relationship between behavioral performance and hemodynamic responses, we conducted Pearson correlation analyses between RT and activation-related features across subjects. Significant correlations were observed for several features in both HbO and HbR concentration changes, and detailed results are summarized in Table 1 and Supplementary Table 1.

**Table 1:** Significant correlations between RT and HbO activation features (*p* < 0.05).

Condition	Feature	*r*	*p*
Target	**Ch12 skewness**	**−0.39**	**0.005**
Nontarget	**Ch01 skewness**	**−0.371**	**0.008**
	Ch02 skewness	−0.339	0.016
	Ch03 kurtosis	−0.323	0.022
	Ch02 slope	0.307	0.03
	Ch03 skewness	−0.303	0.043
	Ch11 kurtosis	0.285	0.044

Pearson correlation coefficients (*r*) and corresponding *p*-values are reported. Bold values indicate nominally significant correlations (*p* < 0.05, uncorrected).

For HbO, significant associations with RT were primarily observed in distribution-related features, including skewness and kurtosis across multiple channels. For instance, skewness in Ch01 (*r* = −0.371, *p* = 0.008) in the nontarget condition and Ch12 (*r* = −0.390, *p* = 0.005) in the target condition showed significant correlations with RT. For HbR, RT was significantly correlated with several features, including slope, skewness, and kurtosis, as summarized in Supplementary Table 1. Slope in Ch02 (*r* = −0.376, *p* = 0.007) in the nontarget condition and skewness in Ch13 (*r* = 0.386, *p* = 0.006) in the target condition were significantly associated with RT. Overall, RT-related correlations were more consistently linked to distributional and temporal features rather than amplitude-based measures.

### 3.3 Activation results

**Figure 3:**
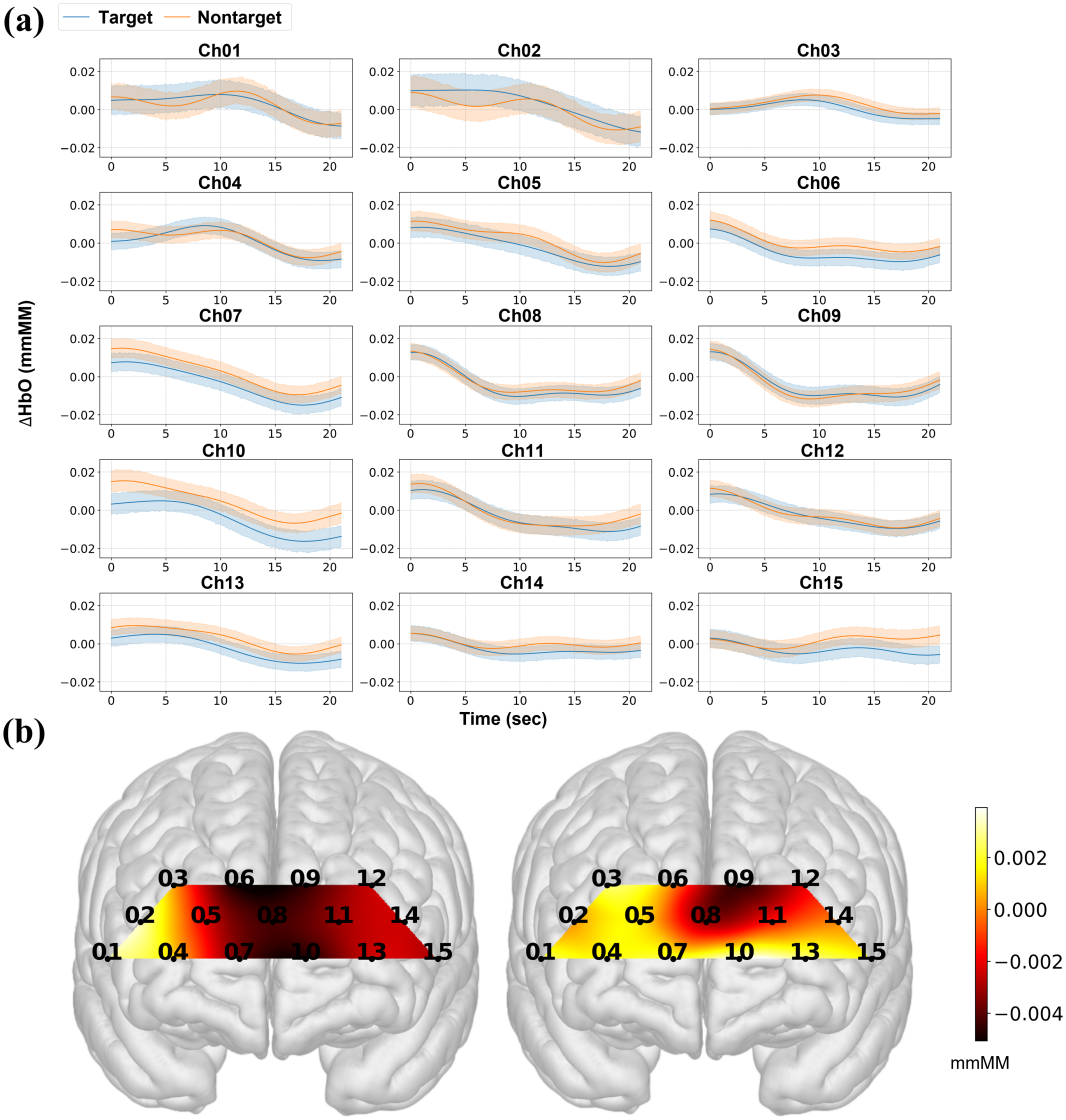
Mean HbO activation changes: **(a)** Time-course of HbO concentration changes over the 21 s analysis window, comprising a 1 s start cue, a 2 s image sequence, and an 18 s rest period following stimulus offset. **(b)** Topographical maps of channel-wise mean HbO activation averaged over the 21 s analysis window (left: target, right: nontarget). Topographical maps were generated by interpolating values between channels for visualization of channel-level distributions.

Hemodynamic activation patterns differed between the target and nontarget conditions across the 21-s analysis window. HbO activation was slightly higher in the nontarget condition than in the target condition, as reflected in both the time-course and topographical maps shown in Figure 3.

**Figure 4:**
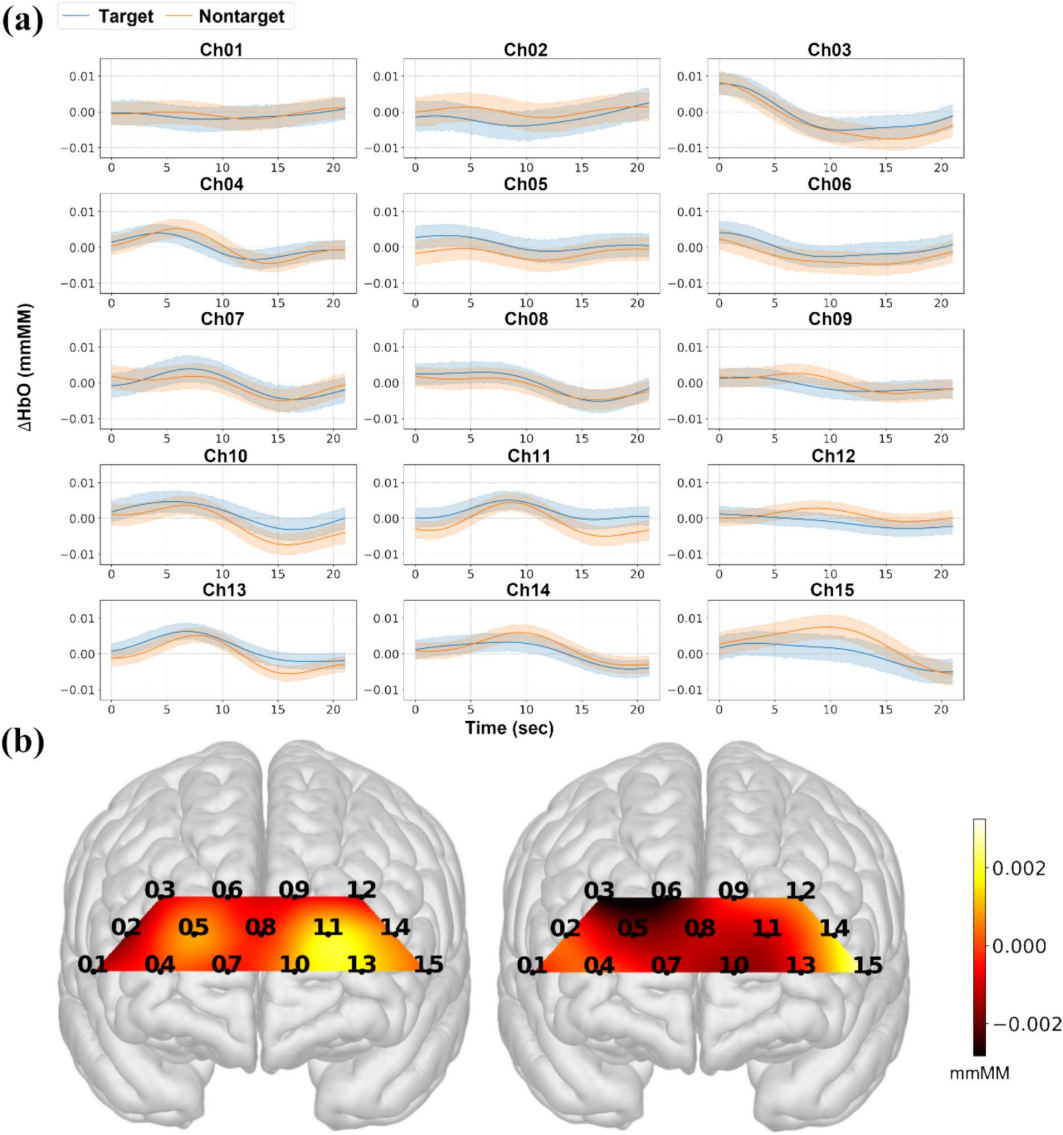
Mean HbR activation changes: **(a)** Time-course of HbR concentration changes over the 21 s analysis window, comprising a 1 s start cue, a 2 s image sequence, and an 18 s rest period following stimulus offset. **(b)** Topographical maps of channel-wise mean HbR activation averaged over the 21 s analysis window (left: target, right: nontarget). Topographical maps were generated by interpolating values between channels for visualization of channel-level distributions.

When averaged across all 15 channels, the mean HbO concentration change was −0.002 (±0.064) for the target condition and 0.001 (±0.061) for the nontarget condition. As shown in Figure 3b, most channels showed greater HbO changes in the nontarget condition. HbR concentration changes showed a slightly different pattern, with higher activation in the mid PFC for the target condition, particularly in Ch05, Ch08, and Ch11, as illustrated in Figure 4.

To quantify these differences, activation-related HbO features were compared between conditions. Table 2 summarizes the HbO features that showed statistically significant differences. Significant effects were observed in several channels, including Ch06, Ch08, Ch10, and Ch13. The largest effect was found in Ch10 for max (*t* = −2,493, *p* = 0.016, d = 0.353), followed by Ch08 for skewness (*t* = −2.423, *p* = 0.019, d = 0.343). Notably, multiple features in Ch10 reached significance, suggesting that this channel may be particularly sensitive to attentional modulation. Across these features, HbO values were generally higher in the nontarget condition, indicating greater or more sustained attentional demand in the absence of a target stimulus.

**Table 2:** Activation-related HbO features showing significant differences between target and nontarget groups (df = 49, *p* < 0.05).

Feature	Target (mean ± SD)	Nontarget (mean ± SD)	*t*-value	*p*-value	Cohen’s *d*
**Ch10 max**	**0.070 (** ± **0.030)**	**0.081 (** ± **0.041)**	**−2.493**	**0.016**	**0.353**
Ch8 skewness	0.040 ( ± 0.145)	0.100 ( ± 0.143)	−2.423	0.019	0.343
Ch10 mean	−0.005 ( ± 0.018)	0.004 ( ± 0.013)	−2.233	0.030	0.316
Ch6 max	0.057 ( ± 0.023)	0.063 ( ± 0.023)	−2.229	0.030	0.315
Ch13 min	−0.064 ( ± 0.027)	−0.057 ( ± 0.024)	−2.113	0.040	0.299
Ch10 min	−0.080 ( ± 0.041)	−0.071 ( ± 0.032)	−2.045	0.046	0.289
Ch10 peak_mean	0.042 ( ± 0.023)	0.050 ( ± 0.026)	−2.040	0.047	0.288

Bold values indicate statistically significant differences after FDR correction (*p* < 0.05).

A similar comparison was performed for HbR features. As shown in Table 3, several features exhibited significant differences between conditions.

**Table 3:** Activation-related HbR features showing significant differences between target and nontarget groups (df = 49, p < 0.05).

Feature	Target (mean ± SD)	Nontarget (mean ± SD)	*t*-value	*p*-value	Cohen’s *d*
**Ch12 kurtosis**	**−0.883 (** ± **0.171)**	**−0.765 (** ± **0.182)**	**−3.817**	**0.000**	**0.540**
Ch15 skewness	0.000 ( ± 0.174)	−0.073 ( ± 0.166)	2.875	0.006	0.407
Ch13 peak_max	0.026 ( ± 0.012)	0.021 ( ± 0.010)	2.321	0.025	0.327
Ch01 skewness	0.050 ( ± 0.173)	−0.005 ( ± 0.185)	2.32	0.033	0.310
Ch13 peak_sum	0.028 ( ± 0.016)	0.022 ( ± 0.012)	2.101	0.046	0.290

Bold values indicate statistically significant differences after FDR correction (*p* < 0.05).

The strongest effect was observed in Ch12 for kurtosis (*t* = −3.817, *p* < 0.001, *d* = 0.540), followed by Ch15 for skewness (*t* = 2.875, *p* = 0.006, *d* = 0.407). These findings indicate that distributional characteristics of the HbR concentration changes were particularly sensitive to task conditions. In contrast to HbO, HbR values were generally higher in the target condition. Effect sizes ranged from small to moderate, suggesting consistent but relatively subtle hemodynamic modulation. Overall, HbO differences were primarily associated with amplitude-related features, whereas HbR differences were more closely linked to distributional characteristics.

### 3.4 Functional connectivity results

We investigated functional connectivity by computing Pearson’s correlation coefficients across all channel pairs to evaluate condition-dependent differences in prefrontal hemodynamic network patterns. Statistical tests with FDR correction revealed significant differences in specific channel pairs, indicating modulation of functional connectivity between conditions. Among the HbO channel pairs, the strongest difference was observed in Ch06-Ch10 (*t* = −3.358, *p* = 0.002, d = 0.475), followed by Ch08-Ch10 (*t* = −2.92, *p* = 0.005, d = 0.413), and Ch08-Ch12 (*t* = −2.493, *p* = 0.016, d = 0.353). Additional significant differences are reported in Table 4.

**Table 4:** Significant channel pairs in HbO functional connectivity between target and nontarget groups (df = 49, *p* < 0.05).

Channel pair	Target (mean ± SD)	Nontarget (mean ± SD)	*t*-value	*p*-value	Cohen’s *d*
**Ch06 – Ch10**	**0.85 (** ± **0.449)**	**0.962 (** ± **0.426)**	**−3.358**	**0.002**	**0.475**
Ch08 – Ch10	0.932 ( ± 0.616)	1.023 ( ± 0.619)	−2.920	0.005	0.413
Ch08 – Ch12	0.785 ( ± 0.480)	0.856 ( ± 0.453)	−2.493	0.016	0.353
Ch07 – Ch12	0.707 ( ± 0.429)	0.796 ( ± 0.423)	−2.492	0.016	0.352
Ch07 – Ch08	1.108 ( ± 0.515)	1.175 ( ± 0.507)	−2.435	0.019	0.344
Ch09 – Ch10	0.917 ( ± 0.561)	0.996 ( ± 0.568)	−2.244	0.029	0.317
Ch10 – Ch12	0.723 ( ± 0.485)	0.803 ( ± 0.511)	−2.229	0.030	0.315
Ch01 – Ch09	0.401 ( ± 0.426)	0.476 ( ± 0.418)	−2.017	0.049	0.285

Pearson correlation coefficients were Fisher’s *r*-to-*z* transformed. Bold values indicate statistically significant differences after FDR correction (*p* < 0.05).

Notably, Ch10 was frequently involved in significant connections, suggesting its central involvement in condition-related connectivity changes.

For HbR concentration changes, the largest effect was found in the Ch05-Ch10 (*t* = 3.577, *p* = 0.001, d = 0.506), followed by Ch06-Ch13 (*t* = 2.596, *p* = 0.012, *d* = 0.367), and Ch01-Ch11 (*t* = −2.57, *p* = 0.013, *d* = 0.363). Additional effects are reported in Table 5.

**Table 5:** Significant channel pairs in HbR functional connectivity between target and nontarget groups (df = 49, *p* < 0.05).

Channel pair	Target (mean ± SD)	Nontarget (mean ± SD)	*t*-value	*p*-value	Cohen’s *d*
**Ch05 – Ch10**	**0.252 (** ± **0.519)**	**0.134 (** ± **0.505)**	**3.577**	**0.001**	**0.506**
Ch06 – Ch13	0.240 ( ± 0.376)	0.165 ( ± 0.357)	2.596	0.012	0.367
Ch01 – Ch11	0.008 ( ± 0.298)	0.116 ( ± 0.395)	−2.57	0.013	0.363
Ch06 – Ch10	0.475 ( ± 0.424)	0.386 ( ± 0.450)	2.524	0.015	0.357
Ch06 – Ch12	0.453 ( ± 0.340)	0.369 ( ± 0.416)	2.238	0.030	0.316
Ch06 – Ch11	0.354 ( ± 0.467)	0.272 ( ± 0.486)	2.091	0.042	0.296

Pearson correlation coefficients were Fisher’s r-to-z transformed. Bold values indicate statistically significant differences after FDR correction (*p* < 0.05).

**Figure 5:**
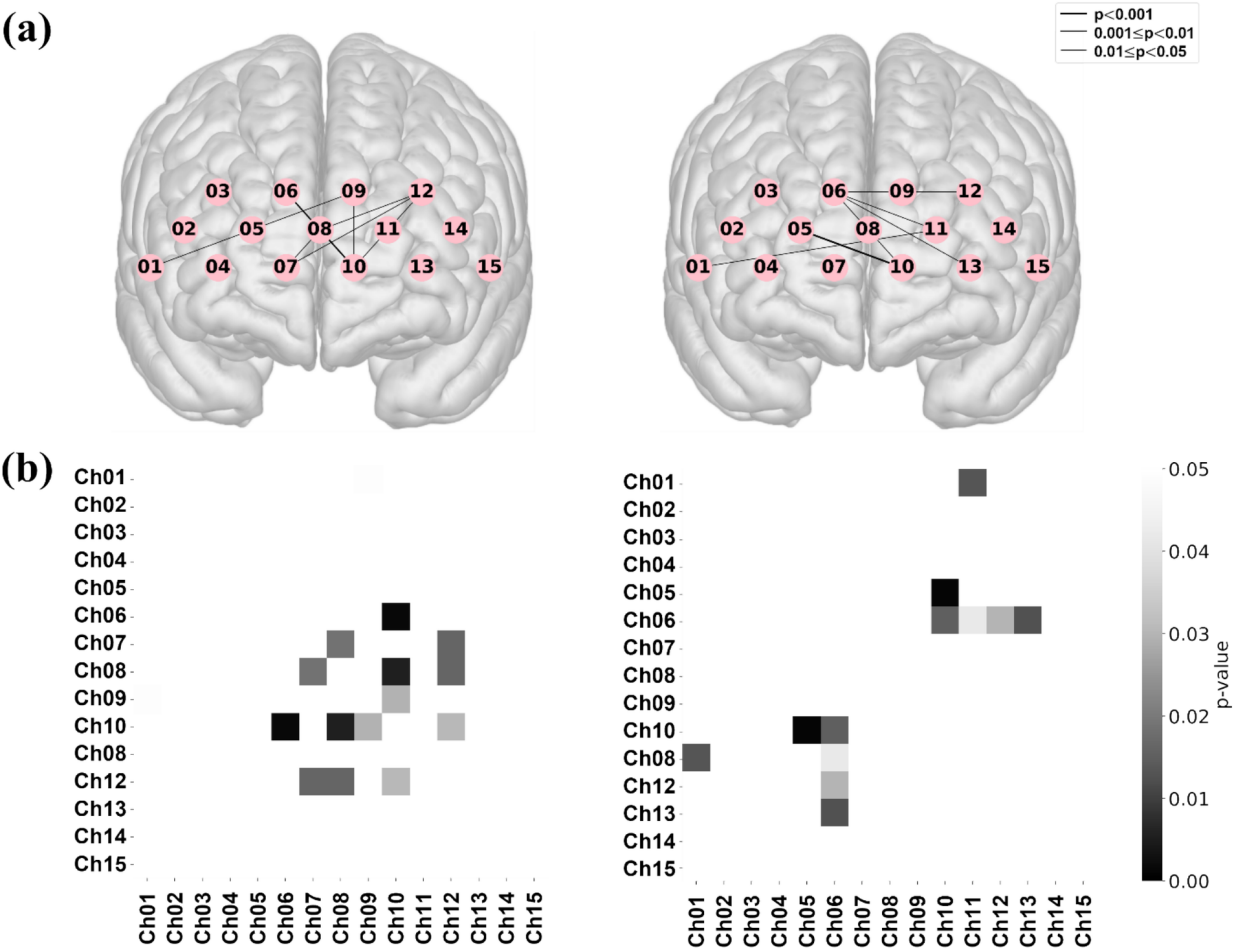
Functional connectivity differences between target and nontarget conditions (left: HbO, right: HbR). **(a)** Significant connectivity network map showing channel pairs with significant differences (*p* < 0.05). **(b)** Significance matrix of all channel pairs (*p* < 0.05).

Several significant connections involved Ch06, indicating its potential role as a key channel in HbR connectivity modulation. The significant connectivity network and corresponding differences are illustrated in Figure 5.

## 4 Discussion

This study investigated condition-dependent differences in PFC hemodynamic responses during RSVP tasks by examining both activation-related features and functional connectivity patterns derived from fNIRS measurements. A key exploratory finding was that the nontarget condition showed a more pronounced HbO-based response pattern, including higher activation magnitude and stronger HbO functional connectivity. In contrast, HbR differences were more closely related to temporal and distributional characteristics of the hemodynamic response. These findings suggest that condition-dependent effects in RSVP tasks may not be captured by a single hemodynamic feature type, but instead emerge from multiple feature domains reflecting both signal magnitude and temporal structure. Overall, these results provide preliminary evidence that prefrontal hemodynamic responses may reflect condition-dependent attentional processing demands under rapid visual presentation conditions.

The nontarget condition showed a relatively stronger HbO response, as reflected by slightly increased HbO levels in the visualization results and higher amplitude-related features, particularly max and mean. Rather than indicating attentional demand directly, this pattern may reflect greater sustained monitoring or decision-related processing in the nontarget condition. In nontarget trials, participants had to evaluate the entire RSVP stream and confirm the absence of a target, whereas target trials provided an explicit target cue that could support the response decision. This interpretation is consistent with prior fNIRS studies showing that prefrontal HbO responses increase with higher task difficulty, cognitive load, and sustained attentional engagement (Hirshfield et al., 2024; Li et al., 2023; Levy and Wagner, 2011; Huang et al., 2015). The longer RT observed in the nontarget condition further supports the possibility that nontarget trials required greater sustained evaluation. HbO differences were primarily characterized by amplitude-related features, with max showing the largest effect size (*d* = 0.353), while mean activation provided additional supporting evidence. The significant skewness difference (*d* = 0.343) further suggests that temporal asymmetry may also contribute to condition-dependent differences, although this effect was secondary to the dominant amplitude-based pattern (Goshvarpour and Goshvarpour, 2023). Thus, HbO responses were characterized mainly by changes in activation magnitude, with a smaller contribution from distributional properties (Lin et al., 2025; Laguë-Beauvais et al., 2013). These effects were most observed at the channel level, particularly involving Ch10, suggesting that this channel contributed consistently to the observed condition-dependent HbO differences. In contrast, HbR concentration changes exhibited a distinct pattern that was more strongly associated with distributional and transient-related features. Kurtosis showed the largest effect size (*d* = 0.540), with lower values in the target condition, indicating that HbR values in the target condition were less influenced by extreme deviations than those in the nontarget condition (Westfall, 2014). Skewness differences were also observed in Ch15 and Ch01, indicating asymmetry in the HbR amplitude distribution (Naseer et al., 2016; Hong and Santosa, 2016). Peak-related features such as peak_mean and peak_sum further reflected differences in peak-level signal characteristics (Ye et al., 2024). Collectively, these findings suggest that HbO and HbR may capture complementary aspects of condition-dependent hemodynamic modulation (Peng and Hou, 2021). While HbO differences primarily reflect changes in overall activation magnitude, HbR responses provide additional information about the shape, variability, and temporal pattern of the hemodynamic response (Machado et al., 2021). These results support the importance of integrating amplitude-based and temporal features to obtain a more comprehensive characterization of PFC activity during RSVP tasks (Novi et al., 2023; Yang et al., 2025).

Building on these activation-level findings, functional connectivity analysis further revealed condition-dependent modulation in inter-channel relationships. In HbO connectivity, the largest difference was observed in the Ch06-Ch10 pair (*d* = 0.475), followed by Ch08-Ch10 and Ch08-Ch12. Several significant HbO connections involved Ch10, suggesting that the observed connectivity differences were partly expressed in repeated channel-level patterns. Consistent with the activation-level findings, HbO connectivity values were generally higher in the nontarget condition, indicating that the nontarget condition was associated not only with higher HbO activation magnitude but also with stronger inter-channel coupling (Cheng et al., 2024; Nguyen et al., 2021; Fishburn et al., 2014; Zeng et al., 2025). For HbR functional connectivity, significant condition-dependent differences were observed across several channel pairs, with the strongest effect found in the Ch05-Ch10 connection (*d* = 0.506), followed by Ch06-Ch13 and Ch01-Ch11. Connectivity values were generally higher in the target condition, indicating stronger inter-channel coupling during target-present trials (Nguyen et al., 2021; Ding et al., 2024). Together, these findings indicate that condition-dependent differences were reflected not only in activation-related features but also in inter-channel coupling among the prefrontal channels (Nguyen et al., 2021; Zeng et al., 2025).

The relationship between hemodynamic patterns and behavioral performance further clarifies this interpretation. RT was significantly longer in the nontarget condition, supporting the view that confirming target absence required more sustained evaluation than detecting target presence (Ayaz et al., 2012; Herff et al., 2014). Correlation analyses showed that RT was more consistently associated with distributional and temporal characteristics of the hemodynamic response, such as skewness, kurtosis, and slope, rather than with amplitude-based measures (Naseer and Hong, 2015; Naseer et al., 2016). Importantly, these RT-associated features did not fully overlap with the features that showed condition-level activation differences, suggesting a partial dissociation between neural signatures of condition effects and those associated with inter-individual variability in behavioral response latency (Naseer et al., 2016). In this exploratory analysis, behavioral response latency appeared to be more closely related to the temporal evolution of the hemodynamic response than to its overall activation magnitude. HbR features also showed a broader range of significant associations with RT than HbO features, suggesting that HbR may provide complementary information about response-related variability in hemodynamic dynamics (Li et al., 2022; Jahani et al., 2017; Yuan and Ye, 2013).

These findings provide further context for the use of prefrontal fNIRS in RSVP-based attentional monitoring. Although the PFC is not the primary cortical region for early visual processing, prefrontal fNIRS may provide additional information about higher-order cognitive processes, including sustained attention, cognitive control, and effort-related regulation (Klein et al., 2024; Fan et al., 2021). The longer RT in the nontarget condition and its association with temporal hemodynamic features suggest that prefrontal fNIRS may be sensitive to sustained processing demands over time (Fan et al., 2021; Harrivel et al., 2013). In addition, prefrontal fNIRS has practical advantages for real-world applications because forehead-based sensor placement can reduce hair interference and improve signal stability (Klein et al., 2024). These properties support its potential utility for monitoring attentional engagement in applied settings, particularly in tasks involving repeated or prolonged cognitive demands (Fan et al., 2021; Harrivel et al., 2013).

Consistent with prior fNIRS-BCI studies reporting the utility of statistical time-domain features, including mean, peak, and slope, for cognitive state classification (Aydin, 2020; Zhang et al., 2022), the present results further suggest that different feature domains capture complementary aspects of attentional processing (Xu et al., 2025). Amplitude-based features, such as mean and max, primarily reflected changes in activation magnitude, whereas distributional and temporal features, including skewness, kurtosis, and slope, captured variations in the shape and temporal dynamics of the hemodynamic response. This distinction suggests that attentional states during RSVP tasks cannot be fully characterized by a single type of feature. Instead, complementary feature representations may be necessary to capture both the strength and temporal structure of prefrontal hemodynamic activity (Xu et al., 2025; Zafar et al., 2023) Future multimodal approaches integrating fNIRS-derived activation and connectivity features with EEG-based indices may further improve the characterization of attentional processing during fast-paced visual tasks, although issues related to temporal alignment, spatial resolution, and task design constraints should be carefully addressed (Xu et al., 2025; Turay et al., 2025; Rizer, 2016).

This study has several limitations. Only young adult women were recruited. This sampling strategy reflected the available participant pool at our institution. Given the exploratory nature of the present study, the relatively homogeneous sample also helped reduce one potential source of inter-subject variability during the initial characterization of condition-dependent prefrontal hemodynamic patterns (Molina-Rodríguez et al., 2025; Wang et al., 2025). However, this restriction limits the generalizability of the findings to broader populations (Kameyama et al., 2004; Xu et al., 2020; Tian et al., 2025). Future studies should include sex-balanced and demographically diverse samples to evaluate the robustness of the observed patterns across participant groups. The effect sizes were also generally small, although some activation and connectivity differences reached statistical significance. Therefore, the findings should be interpreted as preliminary evidence of condition-dependent prefrontal hemodynamic modulation rather than as definitive markers of RSVP attentional states. In addition, the present analysis was conducted at the group level using participant-averaged hemodynamic responses. Although this approach was appropriate for the initial characterization of prefrontal hemodynamic patterns, future work should incorporate single-trial analyses and classification frameworks to evaluate the reliability and predictive utility of the identified features at the individual level (Bauernfeind et al., 2014; von Lühmann et al., 2020; Khan et al., 2024; Benerradi et al., 2023; Zafar et al., 2023).

Further methodological refinements are also needed. Functional connectivity was estimated using zero-lag correlation, and explicit modeling of physiological confounds or hemodynamic delays between regions was not performed. Although short-channel regression and band-pass filtering were applied to reduce superficial and systemic physiological influences (Yücel et al., 2021), residual global or systemic signals may still have affected the correlation-based connectivity estimates. While zero-lag correlation captures overall co-activation patterns commonly examined in fNIRS-based functional connectivity analyses, it may not fully reflect the temporal dynamics of inter-regional interactions (Baker et al., 2018; Medvedev et al., 2011). Future studies should incorporate physiology-informed correction approaches using concurrently recorded systemic signals and employ delay-sensitive methods, such as time-lagged correlation, wavelet coherence, or model-based effective connectivity analyses, to better account for physiological confounds and characterize the temporal structure of functional connectivity (Baker et al., 2018; Li et al., 2022). In addition, broader cortical dynamics cannot be fully captured by prefrontal fNIRS alone, particularly because attentional and working-memory processes may involve distributed fronto-parietal networks (Medvedev et al., 2011; Soekadar et al., 2025). Future studies should therefore investigate how prefrontal hemodynamic responses can be combined with EEG-derived indices reflecting broader and faster neural dynamics, while also improving temporal synchronization across modalities (Pinto-Orellana et al., 2024; Baker et al., 2018; Li et al., 2022). Increasing sample size and task repetitions, accounting for inter-individual variability, and optimizing RSVP paradigms to reduce visual fatigue will also be important for improving reliability and translational applicability (Xu et al., 2025; Medvedev et al., 2011; Su et al., 2023). Building on these exploratory findings, future studies could evaluate the utility of the identified activation and connectivity features using machine-learning or deep-learning approaches for trial-level prediction and fNIRS-based attentional monitoring in fast-paced visual contexts (Xu et al., 2025; Li et al., 2022; Eastmond et al., 2022).

## 5 Conclusion

This study provides an initial exploratory characterization of prefrontal fNIRS hemodynamic responses during RSVP tasks, focusing on both activation-related features and functional connectivity patterns. The results demonstrated that target and nontarget conditions are characterized by complementary hemodynamic signatures. Specifically, HbO differences were primarily associated with changes in activation magnitude, whereas HbR differences reflected variations in the temporal structure and distribution of the response. Functional connectivity analyses further revealed condition-dependent modulation in inter-channel relationships, indicating that attentional processing in RSVP tasks was reflected not only activation-related features but also in inter-channel coupling within the PFC channels. In addition, exploratory correlation analyses showed that RT was more strongly associated with temporal and distributional features of the hemodynamic response than with amplitude-based features. This pattern suggests a partial dissociation between neural processes underlying condition differences and those associated with behavioral response variability. As an exploratory study, these findings systematically identify feature-level and network-level patterns that characterize attentional processing under rapid visual presentation conditions. These findings highlight the importance of integrating multiple feature domains, suggesting that a combination of amplitude-based and temporal features may provide a more comprehensive representation of prefrontal hemodynamic activity. These findings further support the potential utility of prefrontal fNIRS for practical attentional monitoring, particularly when integrated with complementary modalities such as EEG. Overall, this study provides a foundation for future multimodal approaches to fNIRS-based attentional monitoring real-world settings.

## Data Availability

Due to ethical and privacy concerns, the raw data supporting the conclusions of this article are not publicly available. Requests to access the data should be directed to the corresponding author, Suh-Yeon Dong, sydong@sm.ac.kr.
