# Supplementary material for: Prefrontal fNIRS hemodynamic correlates of attentional load during rapid serial visual presentation tasks
Source: Front Hum Neurosci. 2026 Jun 8;20:1843879. doi: 10.3389/fnhum.2026.1843879 (PMC13283837; doi:10.3389/fnhum.2026.1843879)
Supplement: Supplementary file 1 [file Data_Sheet_1.PDF]

# *Supplementary Material*

## 1 Supplementary Figures and Tables

### 1.1 Supplementary Tables

**Supplementary Table 1.** Significant correlations between RT and HbR activation features. Pearson correlation coefficients (r) and corresponding p-values are reported.

| Condition | Feature | r | p |
|---|---|---|---|
| Target | **Ch13 skewness** | **0.386** | **0.006** |
| | Ch02 skewness | 0.366 | 0.009 |
| | Ch11 slope | -0.323 | 0.022 |
| | Ch07 kurtosis | -0.31 | 0.028 |
| | Ch07 mean | 0.299 | 0.035 |
| | Ch09 slope | -0.287 | 0.043 |
| Nontarget | **Ch02 slope** | **-0.376** | **0.007** |
| | Ch07 kurtosis | 0.377 | 0.007 |
| | Ch04 slope | -0.333 | 0.018 |
| | Ch03 kurtosis | 0.331 | 0.019 |
| | Ch14 slope | -0.319 | 0.024 |
| | Ch04 skewness | -0.301 | 0.034 |
